# Salidroside Inhibits CCl_4_-Induced Liver Fibrosis in Mice by Reducing Activation and Migration of HSC Induced by Liver Sinusoidal Endothelial Cell-Derived Exosomal SphK1

**DOI:** 10.3389/fphar.2021.677810

**Published:** 2021-05-13

**Authors:** Qiannan Ye, Yang Zhou, Changqing Zhao, Lieming Xu, Jian Ping

**Affiliations:** ^1^ Shuguang Hospital Affiliated to Shanghai University of Traditional Chinese Medicine, Shanghai, China; ^2^ Institute of Liver Diseases, Shanghai University of Traditional Chinese Medicine, Shanghai, China; ^3^ Yueyang Hospital Affiliated to Shanghai University of Traditional Chinese Medicine, Shanghai, China; ^4^ Shanghai Key Laboratory of Traditional Chinese Medicine, Shanghai, China; ^5^ Key Laboratory of Liver and Kidney Diseases, Ministry of Education, Shanghai, China

**Keywords:** salidroside, liver fibrosis, SphK/S1P/S1PRs, exosomes, liver sinusoidal endothelial cells, hepatic stellate cell

## Abstract

Sphingosine kinase 1 (SphK1)/Sphingosine-1-phosphate (S1P)/S1PRs signaling pathway is known to involve the advancement of liver fibrosis. Exosomal SphK1 promotes hepatic stellate cells (HSC) migration. Salidroside (Sal) inhibits liver fibrosis, but its mechanism is yet to be elucidated. This study was to explore the influences of Sal on the SphK/S1P/S1PRs signaling pathway in liver fibrosis induced by carbon tetrachloride (CCl_4_) *in vivo*, and investigated the mechanism of Sal affecting the migration and activation of HSC triggered by exosomal SphK1 *in vitro*. Our data showed that Sal reduced the activities of alanine transaminase (ALT), aspartate aminotransferase (AST) in serum, and hydroxyproline (Hyp) content in the liver tissue. Sal subdued the expression of α-smooth muscle actin (α-SMA), fibronectin (FN) and type I collagen (Col I) of the liver. Sal also reduced mitochondria-induced hepatocyte apoptosis and to inhibit JNK activation. Furthermore, Sal remarkably eradicated the influence of SphK1, SphK2, S1P, and S1PRs triggered by CCl_4_, whether stimulating or hindering. Compared with serum-derived exosomes from model group mice, serum-derived exosomes from Sal group mice expressed lower SphK1 and reduced JS 1 (mouse HSC cell line) migration. In addition, Sal was also observed to subdue Col I expression, AKT activation, and LX-2 migration induced by exosomal SphK1 from SK-HEP-1 (a kind of liver sinusoidal endothelial cells (LSEC) cell line). In conclusion, Sal could effectively alleviate liver injury, hepatocyte apoptosis, and liver fibrosis *in vivo*, providing supports that the protective effects of Sal might be realized by suppressing JNK activation and modulating the SphK/S1P/S1PRs axis. *In vitro*, it was observed that Sal might alleviate LX-2 migration and activation induced by exosomal SphK1 by inhibiting the AKT activation.

## Introduction

Liver fibrosis involves excessive accumulation of collagen and extracellular matrix (ECM). It is a reversible over-repair process of liver tissue damage due to various chronic liver injuries (Hernandez-Gea and Friedman, 2011). Hepatic stellate cell (HSC) activation is supposedly the critical event that initiates and develops liver fibrosis (Tsuchida and Friedman, 2017), though the exact pathogenesis is yet to be understood. Current effective treatment for liver fibrosis involves etiological treatment, such as antiviral therapy, the use of immunosuppressive agents, and others. This calls for researching effective therapies for alleviating liver fibrosis.

Exosomes are small vesicles released by various cells and distributed in several body fluids. Exosomes can mediate information exchange among cells and are a part of numerous physiological and pathological activities, including cell growth, proliferation, differentiation, and apoptosis (van Niel et al., 2018). Hepatocytes and liver sinusoidal endothelial cells (LSECs) have been shown in recent investigations to interact with HSCs via exosomes, in turn regulating the biological activity of HSCs (Sung et al., 2018). For instance, the miR-192 content in exosomes from palmitic acid-stimulated hepatocytes was found to be considerably increased, and these exosomes stimulated the activation of HSCs (Lee et al., 2017). LSECs secrete exosomes that express high amounts of Sphingosine kinase 1 (SphK1), which promote HSC migration by triggering AKT phosphorylation (Wang et al., 2015).

SphK/Sphingosine-1-phosphate (S1P)/S1PRs signaling pathway participates in tissue fibrosis progression, including liver fibrosis (Kono et al., 2007; Sato et al., 2016; Wang et al., 2019). The renal tissues of mice with unilateral ureteral ligation-caused renal interstitial fibrosis, the SphK1 levels, and autophagy-related proteins were found to have remarkably amplified as fibrosis advanced. The SphK1 protein expression was also detected to be upregulated in the lung tissue of bleomycin-induced pulmonary fibrosis model mice. In addition, when the SphK1 expression was blocked by SphK inhibitors or siRNA, the high expression of α-smooth muscle actin (α-SMA) and fibronectin (FN) induced by transforming growth factor β1 (TGF-β1) was observed to be lowered (Kono et al., 2007). Likewise, the SphK1 expression and its product S1P were recorded to increase progressively as the liver fibrosis advanced in the bile duct ligation (BDL) and carbon tetrachloride (CCl_4_)-induced liver fibrosis, mouse model. Furthermore, S1P promoted liver fibrosis angiogenesis via binding to its ligands. Nevertheless, obstructing the binding of S1P to ligands perhaps inhibits angiogenesis in fibrotic liver tissue, in turn lowering the degree of liver fibrosis (Yang et al., 2013). These findings indicate that SphK/S1P/S1PRs signaling pathway plays an essential role in organ fibrosis.

A phenolic compound, Salidroside (Sal), occurs in plants of *Rhodiola rosea* L. Sal has been reported in recent researches to possess several pharmacological properties, such as anti-inflammatory (Xu et al., 2019), antioxidative (Jiansheng and Ying, 2012), and anticancerous (Ren et al., 2019). Earlier researches evidenced Sal to exhibit curative effects on numerous liver diseases. For instance, Sal reduces drug-induced liver injury (Yuan et al., 2019), guards the liver against ischemia/reperfusion injury (Cai et al., 2017), and eases nonalcoholic fatty liver disease (NAFLD) by AMPK-dependent TXNIP/NLRP3 pathway (Zheng et al., 2018). Another study reported Sal obstructing HSC activation and autophagy by downregulating NF-κB and TGF-β1/Smad3 signaling pathway (Feng et al., 2018), though it is unclear whether the effect of Sal on liver fibrosis is correlated to the SphK/S1P/S1PRs signal pathway. In one of our earlier investigations, Sal was observed to hinder the HSCs migration by obstructing AKT phosphorylation (Zhang et al., 2012). Based on literature reports evidencing exosomes to overexpress SphK1 derived from LSEC promoted LX-2-migration by upregulating the AKT activation, Sal was theorized to subdue HSC migration and activation by regulating exosome-mediated communication between LSEC and HSC.

The effects of Sal on SphK/S1P/S1PRs signal pathway in mouse with liver fibrosis caused by CCl_4_ and how it regulates the migration and activation of LX-2 induced by exosomal SphK1 from LSECs were explored in this study *in vivo* and *in vitro*, respectively. The outcomes support the efficacy of Sal as an antifibrotic drug and back the SphK/S1P/S1PRs efficiency as a therapeutic target for liver fibrosis.

## Materials and Methods

### Materials

Sal was procured from Shanghai Tauto Biotechnology (Shanghai, China), the alanine transaminase (ALT), aspartate aminotransferase (AST), and hydroxyproline (Hyp) detection kits were acquired from Nanjing Jiancheng Bioengineering Institute (Nanjing, China). FN, type I collagen (Col I), Bax and Bcl-2 antibodies were obtained from Abcam (Cambridge, United Kingdom). JNK, p-JNK, PARP, and cleaved caspase 3 antibodies were purchased from Cell Signaling Technology (Massachusetts, United States). SphK1, SphK2, S1PR1, S1PR2, S1PR3, CD9, and tumor susceptibility gene 101 (TSG101) antibodies were procured from ImmunoWay Biotechnology Company (Suzhou, China). Antibody against GAPDH was provided by Proteintech (Wuhan, China). TdT-mediated dUTP nick end labeling (TUNEL) Apoptosis Assay Kit, 2-(4-Amidinophenyl)-6-indolecarbamidine dihydrochloride (DPAI), and Cy3-labeled goat anti-rabbit IgG were all bought from Beyotime Institute of Biotechnology (Shanghai, China). The S1P Assay Kit (S1P-ELISA) was delivered by Echelon Biosciences (Salt Lake City, United States).

### Animals

Male C57BL/6J, SPF grade mice weighing 20 ± 2 g, were supplied by Shanghai Sippr-BK laboratory animal Co. Ltd. (Shanghai, China). They were placed in the experimental animal center of Shanghai University of Traditional Chinese Medicine, China. The Committee on the Care and Use of Live Animals for Teaching and Research of the Shanghai University of Traditional Chinese Medicine sanctioned the animal experiments.

### Animal Treatments

Following a week-long adaptive feeding, 45 male mice were divided into the control group included 15 mice, while the modeling group had 30. The modeling group animals were intraperitoneally injected with 15% CCl_4_ (diluted with olive oil) at 2 ml/kg thrice a week, whereas those in the control group were administered an equal dosage of olive oil intraperitoneally. After a week of modeling, the modeling group was segregated into Model and Sal groups. The Model and Sal groups were continued to model with CCl_4_ for 5 weeks, while the Control group was injected intraperitoneally with equal dose of olive oil. The Sal group animals were administered Sal (20 mg/kg) through gavage for 5 weeks. Simultaneously, those in the Control and Model groups were both treated with equal doses of ddH_2_O. At the end of five weeks, the mice were sacrificed on anesthetizing with pentobarbital sodium, and the serum and liver tissue samples were preserved for subsequent experiments.

### Serum Biochemical Test

The instructions provided along with the kit were followed to detect ALT and AST levels in the mice serum.

### Pathological Evaluation of Liver

Fixation of the liver tissues was carried out in 10% neutral formalin for 48 h. They were then dehydrated stepwise with ethanol, followed by xylene permeation, and finally paraffin-embedded. Sections of 4 μm thickness were prepared for HE and Sirius red staining.

### Determination of Hyp Content

Following the kit directions, the Hyp contents in liver tissues were determined.

### Human Samples

Healthy volunteers and cirrhosis patients were selected for collecting human liver tissue and serum samples from the Department of Hepatobiliary Surgery, Renji Hospital, Affiliated to Shanghai Jiao Tong University. The Ethics Committee of Shuguang Hospital, Affiliated to Shanghai University of Traditional Chinese Medicine, sanctioned this study.

### Isolation of Primary Liver Sinusoidal Endothelial Cells From Mice

Wild and Model groups of five male mice each were formed. The Model group mice were injected with CCl4 intraperitoneally to create a liver fibrosis model. They were then used to isolate primary LSECs. Using density gradient centrifugation in combination with CD146 magnetic bead (Miltenyi Biotec, Germany) separation, the primary LSEC of mice was isolated as the earlier reports (Liu et al., 2011; Meyer et al., 2016). Mouse liver tissue was perfused *in situ* and digested using an enzyme solution of collagenase D and DNase (Roche, Germany). Density gradient centrifugation was performed using OptiPrep solution (Axis-Shield, Norway) and incubation with CD146 magnetic bead for 30 min at 4°C. Thus isolated LSECs were cultured in Col I-coated dishes along with ECM medium, comprising 5% FBS, 2% endothelial cell growth supplement, and maintained with 5% CO_2_ at 37°C in a humidified incubator.

### Cell Culture

Propagation of human immortalized HSCs (LX-2) and mouse immortalized HSCs (JS 1) was carried out in DMEM, supplemented with 10% FBS, 100 U/ml penicillin, and 0.1 mg/ml streptomycin. The SK-HEP-1 cells, obtained from the Chinese Academy of Sciences, were grown in MEM, containing 10% FBS, 100 U/ml penicillin, and 0.1 mg/ml streptomycin. All cell lines were maintained with 5% CO_2_ in a humidified incubator at 37°C.

### Cell Transfection

The lentiviral vectors containing human SphK1 (LV-SphK1) or empty plasmid (LV-Cr) were procured from HANBIO Biotech (Shanghai, China). A 24-well plate was used to culture SK-HEP-1 to a confluence of 30–50%. They were then cultured in a fresh medium containing lentiviral vectors (MOI = 30) and polybrene (5 μg/ml) for 24 h, and then in a fresh medium for further culturing, followed by incubation for 48 h. The transfection efficiency of cells was studied using an inverted microscope and were incubated with a medium containing puromycin (1 μg/ml) to eliminate the untransfected cells. The transfected cells were cultured further, and the culture medium was used to isolate exosomes.

### Isolation of Exosomes

SK-HEP-1 cells were cultured in 150 mm dishes to a confluence of 80–90%. They were then cultured in MEM with 10% exosome-free serum for 24 h. The supernatant was ultracentrifuged to obtain the exosomes. The supernatant was first centrifuged at 4°C, 300 ×g, for 10 min to eliminate cells. The supernatant was centrifuged again at 4°C, 2,000 ×g*,* for 10 min to eradicate dead cells. The supernatant was centrifuged at 4°C, 10,000 ×g*,* for another 30 min to eliminate cell debris. Eventually, the supernatant was ultracentrifuged at 4°C, 100,000 ×g for 70 min, and the precipitate collected was the exosomes. The thus isolated exosomes were resuspended in PBS or DMEM medium for subsequent *in vitro* studies.

### Identification of Exosomes

A transmission electron microscope was utilized to study the morphology of isolated exosomes (van Niel et al., 2018). This was followed by detecting typical exosomal marker proteins, CD9 and TSG101, by Western blot (WB) analysis.

### Cell Treatment

LX-2 and JS 1 were cultured in 60 mm dishes to a confluence of 80–90%. LX-2 cells were allocated to Cr-exo and Cirrhosis-exo groups and incubated with serum exosomes (30 μg/ml) from healthy volunteers and cirrhosis patients, respectively, to study the migration of LX-2 cells. Also, JS 1 cells were distributed into Cr-exo, M-exo, and Sal-exo groups and incubated with serum exosomes (30 μg/ml) from corresponding mice to detect JS 1 cells’ migration. The LX-2 cells were then divided into Cr-exo, SphK1^+^-exo, Cr-exo + Sal, and SphK1^+^-exo + Sal groups and incubated with 30 μg/ml exosomes of transfected SK-HEP-1 cell culture media and/or Sal (10^−5^ M) to study the LX-2 cells migration and activation.

### Transwell Migration Assays

LX-2 or JS 1 cells were cultured in Col I-coated migration cups to a confluence of 80–90%. Then, exosomes and/or Sal were added to the lower chamber, and with the same medium being added to the upper chamber as well. Incubation was carried out for 24 h, and the migration cups were then taken out and air-dried. Fixation of the cells on the lower surface of the migration cup was done using 4% paraformaldehyde and staining with crystal violet. The total number of cells that migrated to the bottom was recorded with an inverted microscope (Olympus, Tokyo, Japan) and evaluated using ImageJ software (NIH, United States).

### Di-Acetylated-Low-Density Lipoprotein Assays

The adhered LSECs were incubated in ECM medium comprising DiI-Ac-LDL (Yiyuan Biotech, Guangzhou, China) for 4 h in the dark. The cells were then rinsed thrice with PBS, fixed with 4% paraformaldehyde for 15 min, and rinsed again with PBS. The endocytosis of DiI-Ac-LDL by LSECs was studied using an inverted fluorescence microscope (Olympus, Tokyo, Japan).

### TUNEL Assay

According to the manufacturer's instructions, sections of frozen liver tissue of 8 μm thickness were prepared and incubated with TUNEL.

### Immunofluorescence Staining

The LSECs were collected following 3 days of culturing, rinsed with PBS, and fixed using 4% paraformaldehyde for 30 min at room temperature. The frozen sections of mice liver tissue were fixed with precooled acetone for 15 min. Once fixed, the cells and frozen sections were rinsed again with PBS and incubated using 0.1% X-Triton-100 for 15 min at room temperature to disrupt the membrane. They were permeabilized and blocked using 5% BSA at room temperature for 1 h and then incubated with respective primary antibodies at 4°C overnight. The following day, they were incubated with anti-rabbit IgG (Cy3-labeled) in the dark at room temperature for 1 h and rinsed again with PBS. The cells were then stained using DAPI and rinsed using PBS. The sample was sealed, and their fluorescence was studied with the help of an inverted fluorescence microscope.

### Protein Isolation and Western Blotting Analysis

RIPA lysate comprising complete Mini, PMSF, and phosphatase inhibitors was utilized to isolate liver tissues and cells' total protein. Protein quantification was performed with the BCA protein assay kit (Beyotime Institute of Biotechnology, Shanghai, China). An equivalent amount (30 μg) of proteins was electrophoresed by SDS-PAGE and transferred to PVDF membranes (Millipore, United States). The membranes were then incubated with blocking buffer (Beyotime Institute of Biotechnology, Shanghai, China) at room temperature for 1 h and incubated with respective primary antibodies overnight at 4°C. The membranes were rinsed using PBS-T for 20 min and treated with anti-rabbit IgG or anti-mouse IgG for 1 h. The membranes were rinsed with PBS-T again, and the images were recorded by means of Odyssey imaging system (LI-COR, United Kingdom) and analyzed with the ImageJ software. A part of the membranes was also stained with ponceau after incubating with antibodies.

### RNA Isolation and RT-PCR Analysis

TRIzol total RNA preparation kit (Shanghai Sangon Biotech, Shanghai, China) was utilized to isolate the total RNA of cells and synthesized to cDNA with a ReverAid First Strand cDNA Synthesis Kit (Thermo, United States) following the manufacturer’s procedures. The cDNA was mixed with primers and SYBR Green Real-time PCR Master Mix (TOYOBO, Japan) in a certain ratio; the relative expressions of mRNA were measured utilizing the ViiA 7 Real-Time PCR System (ABI, Carlsbad, CA, United States). Primer sequences used for detection of Sphk1, SphK2, S1PR1,2,3 were shown in Table 1.

**TABLE 1:** Primer sequences for PCR.

Gene	Forward (5′-3′)	Reverse (5′-3′)
SphK1^a^	TGA​CTT​GGA​AAG​AAA​GGC​TCT	GCA​ATG​GGG​AGT​GTC​TTC​TAT
SphK2^a^	GCACGGCGAGTTTGGTTC	GAG​ACC​TCA​TCC​AGA​GAG​ACT​AG
S1PR1^a^	TGA​GCG​AGG​CTG​CTG​TTT​C	AGA​GGG​CGA​GGT​TGA​GTG​AG
S1PR2^a^	CCT TGA CTG GCT TGA ACT TTC	ACC​CTC​AGA​ACA​CAG​ACA​GGT
S1PR3^a^	ACC​AAG​AAA​ATG​TCA​CCG​TGT	ACT​CCC​CTA​CAC​ACT​GCT​CTG
SphK1^b^	CGA​CGA​GGA​CTT​TGT​GCT​AGT	AAA​CAT​CTC​ACT​GCC​CAG​GT
GAPDH^b^	AGC​CAC​ATC​GCT​CAG​ACA​CC	GTA​CTC​AGC​GCC​AGC​ATC​G

a Mouse

b human.

### Statistical Analysis

The outcomes have been recorded in terms of mean ± standard error of the mean. The differences among groups were evaluated via *t*-test or One-way ANOVA using SPSS 21.0 software. Values of *p* < 0.05 were considered statistically significant.

## Results

### Sal Alleviated CCl_4_-Induced Liver Fibrosis in Mice

**FIGURE 1:**
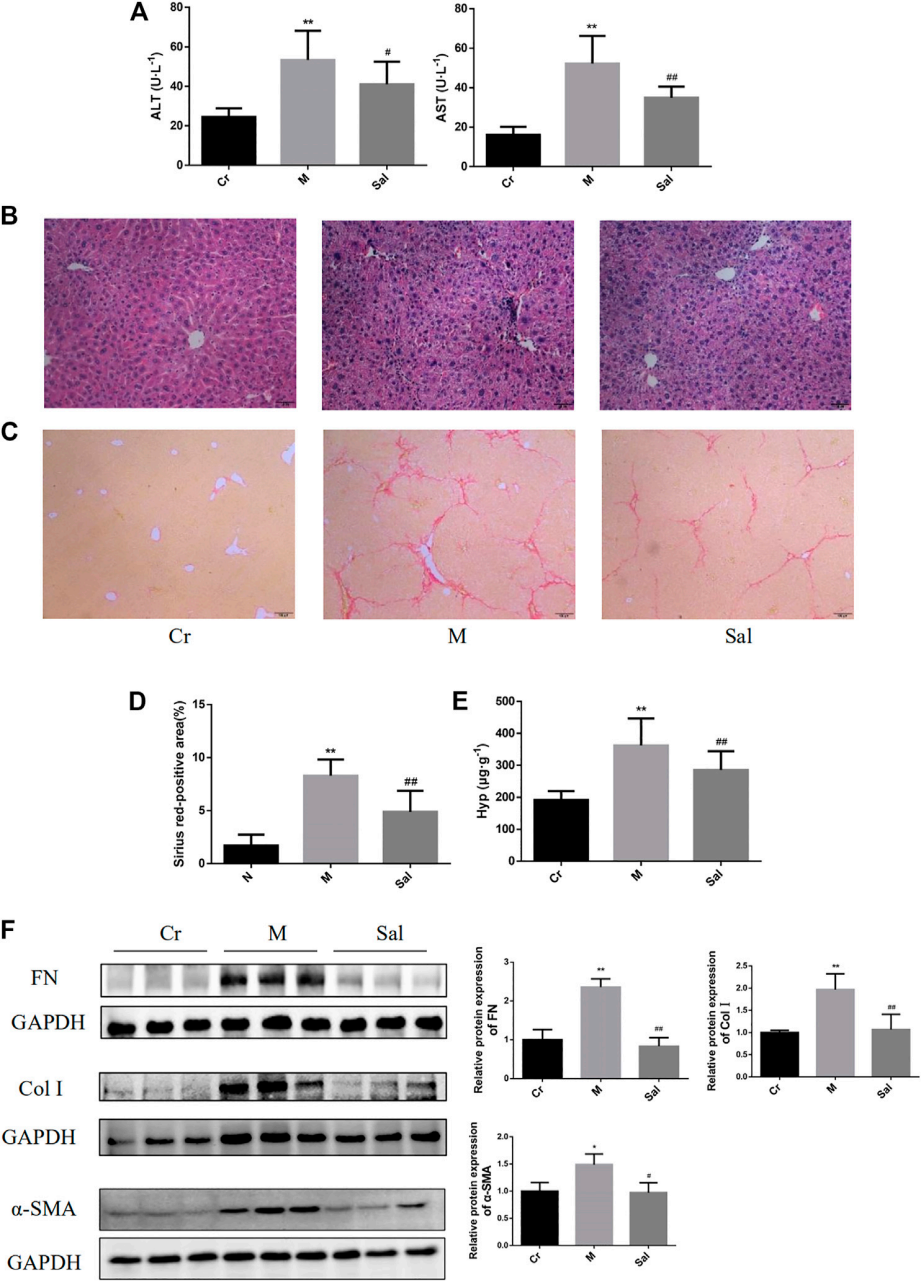
Effects of Sal on liver injury and fibrosis induced by CCl_4_. **(A)**: Levels of serum ATL and AST in different groups. **(B)**: HE staining of liver tissues in each group (×100). **(C)**: Sirius red staining of liver tissue sections (×100). **(D)**: Sirius red-positive area. **(E)**: Contents of Hyp in liver tissue. **p < 0.01, vs. Cr; ^##^ *p* < 0.01, vs. M. **(F)**: Expressions of FN, Col I, and α-SMA in mice liver tissue were detected by WB. (*n* = 15, *p < 0.05, **p < 0.01, vs Cr; ^#^ *p* < 0.05, ^##^ *p* < 0.01, vs M.) ALT: alanine transaminase; AST: aspartate aminotransferase; Hyp: hydroxyproline; Cr: control group; M: model group; Sal: salidroside group. α-SMA: α-smooth muscle actin; FN: fibronectin; Col I: type I collagen; Cr: control group; M: model group; Sal: salidroside group.

Serum ATL and AST were detected to be considerably higher in the Model group than those in the Control group (*p* < 0.01) (Figure 1A), indicating obvious liver function damage due to CCl_4_. Nevertheless, these changes were observed to be reversed by Sal (*p* < 0.05 or *p* < 0.01) (Figure 1A). HE staining revealed that CCl_4_ resulted in collagen deposition, structural destruction, and inflammatory cell infiltration in liver lobules and portal areas. Sal treatment was found to remarkably alleviate these histopathological lesions (Figure 1B).

Besides, Sirius red staining revealed excessive collagen deposition in the Model group than that in the control group, and Sal was noted to lower the collagen deposition (Figures 1C,D) substantially. Then, the effect of Sal on liver fibrosis was further examined by identifying the Hyp content in liver tissue and the characteristic indicators of liver fibrosis, including FN, Col I, and α-SMA. The Hyp content in liver tissues of the Model group was found to be remarkably higher as compared with that of the control group. Sal treatment reduced the Hyp level (*p* < 0.01) (Figure 1E). Moreover, higher quantities of FN, Col I, and α-SMA proteins were expressed in the Model group, which were considerably subdued by Sal administration (*p* < 0.05 or *p* < 0.01) (Figure 1F), signifying Sal reduced CCl_4_-induced liver fibrosis.

### Sal Alleviated CCl_4_-Induced Apoptosis of Hepatocytes in Mice via Regulating JNK Activation

**FIGURE 2:**
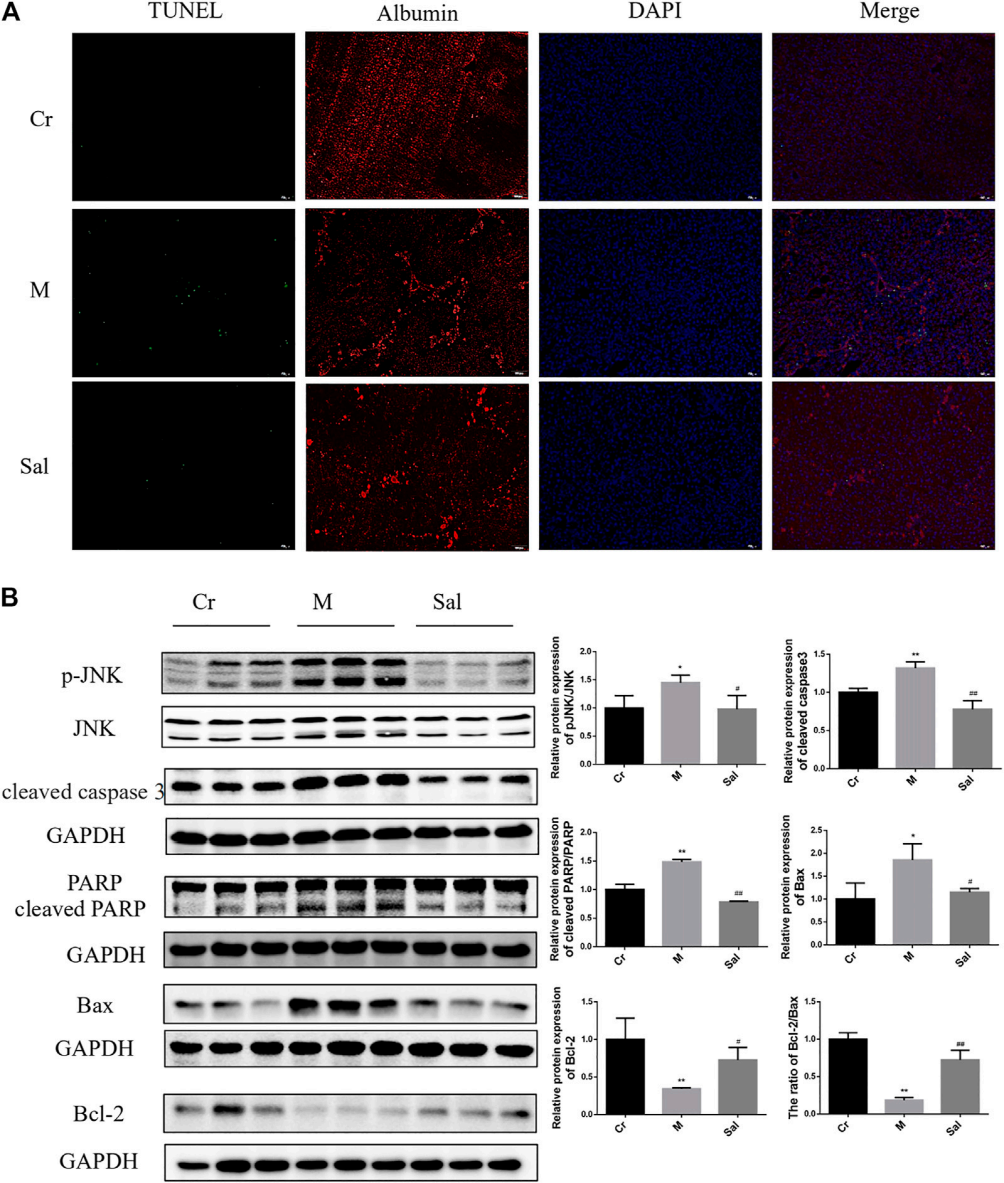
Effect of Sal on apoptosis of hepatocytes in mice with liver fibrosis induced by CCl_4_. **(A)**: Immunofluorescence staining of TUNEL and Albumin (×100). **(B)**: Effects of Sal on the expression of p-JNK, cleaved caspase3, cleaved RARP, Bax, and Bcl-2 in mice liver induced by CCl_4_. (*n* = 15, *p < 0.05, **p < 0.01, vs. Cr; ^#^ *p* < 0.05, ^##^ *p* < 0.01, vs. M.). DAPI: 4′,6-diamidino-2-phenylindole; TUNEL: TdT-mediated dUTP nick end labeling.

Considering the defensive effect of Sal on liver injury and fibrosis, Sal’s influence on hepatocyte apoptosis was further studied by TUNEL staining. As indicated in Figure 2A, the apoptotic hepatocytes in the Model group were significantly amplified compared with those in the control group. Apoptotic cells in the Sal group as compared with the Model group were observed to be considerably reduced, indicating Sal’s efficacy in alleviating CCl_4_-induced hepatocyte apoptosis.

Sal’s efficiency in expressing classic proteins in the mitochondrial apoptosis pathway and the role of the JNK signaling pathway in CCl_4_-induced liver fibrosis were also investigated. Higher levels of cleaved caspase 3, cleaved PARP and Bax were expressed in the Model group, while Bcl-2 was lowered. Sal therapy was detected to reverse these changes and upregulate the ratio of Bcl-2 to Bax (*p* < 0.05 or *p* < 0.01) (Figure 2B). As displayed in Figure 2B, CCl_4_ supported JNK phosphorylation in liver tissues. Sal therapy was recorded to downregulate the enhanced expression of p-JNK in model mice (*p* < 0.05) (Figure 2B). These findings indicate Sal considerably reduced mitochondria-mediated hepatocytes apoptosis by inhibiting JNK phosphorylation.

### Sal Regulated the SphK/S1P/S1PR Signaling Pathway in the Liver of Mice

**FIGURE 3:**
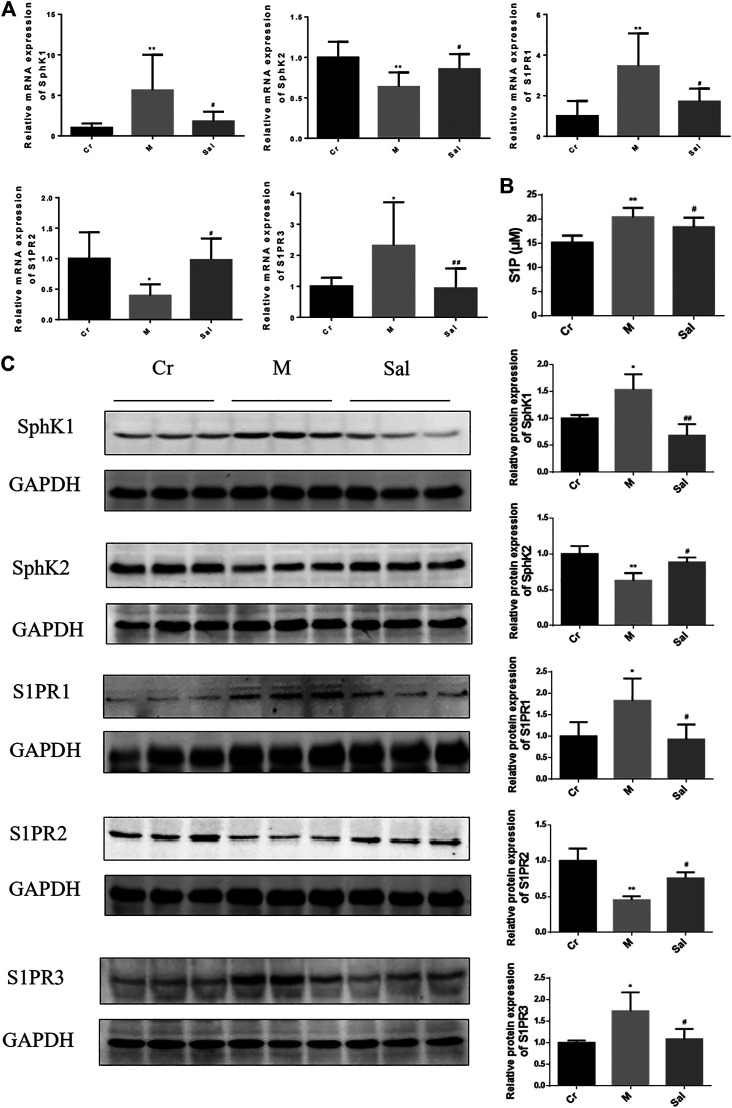
Effects of Sal on the expressions of SphK/S1P/S1PRs signaling pathway in mice liver induced by CCl_4_. **(A)**: The mRNA expressions of SphK1, SphK2, SPPR1, S1PR2 and S1PR3 in mice liver tissue. **(B)**: Level of S1P in serum from mice. **(C)**: The protein expressions of SphK1, SphK2, of S1PR1, S1PR2, and S1PR3 in mice liver tissue (*n* = 15, *p < 0.05, **p < 0.01, vs Cr; ^#^ *p* < 0.05, ^##^ *p* < 0.01, vs. M.) SphK1: Sphingosine kinase 1; S1P: Sphingosine-1-phosphate; S1PR1: Sphingosine 1-phosphate receptor 1; S1PR2: Sphingosine 1-phosphate receptor 2; S1PR3: Sphingosine 1-phosphate receptor 3.

Sal's antifibrosis efficacy in relation to the regulation of SphK/S1P/S1PR signaling pathway was studied by examining the expression levels of SphK, S1P, and S1PRs. The protein and mRNA levels of SphK1 were detected to be upregulated, and SphK2 protein and mRNA expression downregulated in the liver tissues of the Model group as compared with those in the control group (*p* < 0.05 or *p* < 0.01) (Figures 3A,C). Sal was found to inhibit these effects (*p* < 0.01) (Figures 3A,C). Moreover, a high S1P level was observed in the model group (*p* < 0.01) (Figure 3B), and Sal was found to lower its level.

In addition, CCl_4_ was also detected to regulate S1PRs, the receptors of S1P. Notably, it also amplified the expression of S1PR1 and S1PR3 but subdued that of S1PR2. Sal substantially eradicated the effects of S1PRs triggered by CCl_4_, whether stimulating or hindering (Figure 3C).

### Sal Inhibited the Expression of SphK1 in Serum Exosomes and the Migration of HSC Induced by Serum Exosomes

**FIGURE 4:**
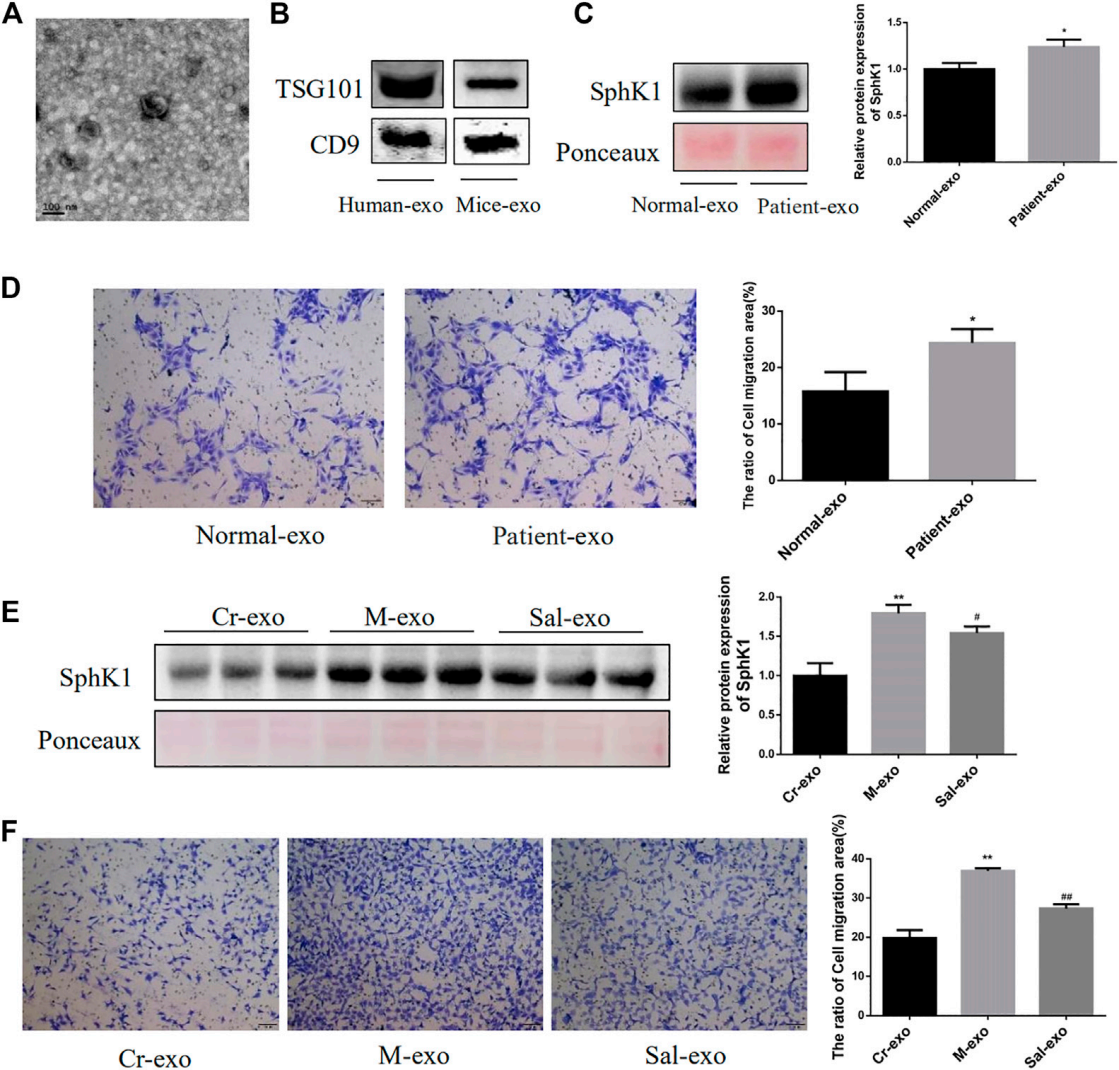
Characteristics of serum exosomes and their effect on migration of HSC. **(A)**: Serum exosomes had a double-layer membrane structure. **(B)**: Expression of TSG101 and CD9 in serum exosomes from human and mice. **(C)**: Expression of SphK1 in serum exosomes from human (*n* = 6, *p < 0.05, vs. Normal-exo.) **(D)**: Effects of serum exosomes from different populations on LX-2 migration (*n* = 6, *p < 0.05, vs Normal-exo.) **(E)**: Expression of SphK1 in serum exosomes from different groups of mice (*n* = 15, **p < 0.01, vs. Cr-exo, ^#^ *p* < 0.05, vs. M-exo,) **(F)**: Effects of serum exosomes from different groups of mice on JS 1 migration (*n* = 15, **p < 0.01, vs Cr-exo, ^##^ *p* < 0.01, vs. M-exo.) TSG101: tumor susceptibility gene 101.

Serum exosomes from mice and humans were successfully isolated in this research, and their characteristics were analyzed by electron microscopy (Figure 4A), while the expression of markers of exosomes, TSG101 and CD9, was identified by Western blot (Figure 4B). SphK1 was noted to be highly expressed in serum exosomes from liver cirrhosis patients, which encouraged the migration of LX-2 (*p* < 0.01) (Figures 4C,D). A similar remarkably high expression of SphK1 in the serum exosomes derived from mice with liver fibrosis was recorded (*p* < 0.01) (Figure 4E). Moreover, Sal treatment was detected to downregulate the expression of SphK1 in serum exosomes (*p* < 0.05) (Figure 4E). As theorized, the serum exosomes in the mice with liver fibrosis were also found to encourage the migration of JS 1, and the serum exosomes of the Sal group to subdue the migration of JS 1 (*p* < 0.01) (Figure 4F).

### SphK1 was Highly Expressed in Primary LSEC of Mice With Liver Fibrosis Induced by CCl_4_

**FIGURE 5:**
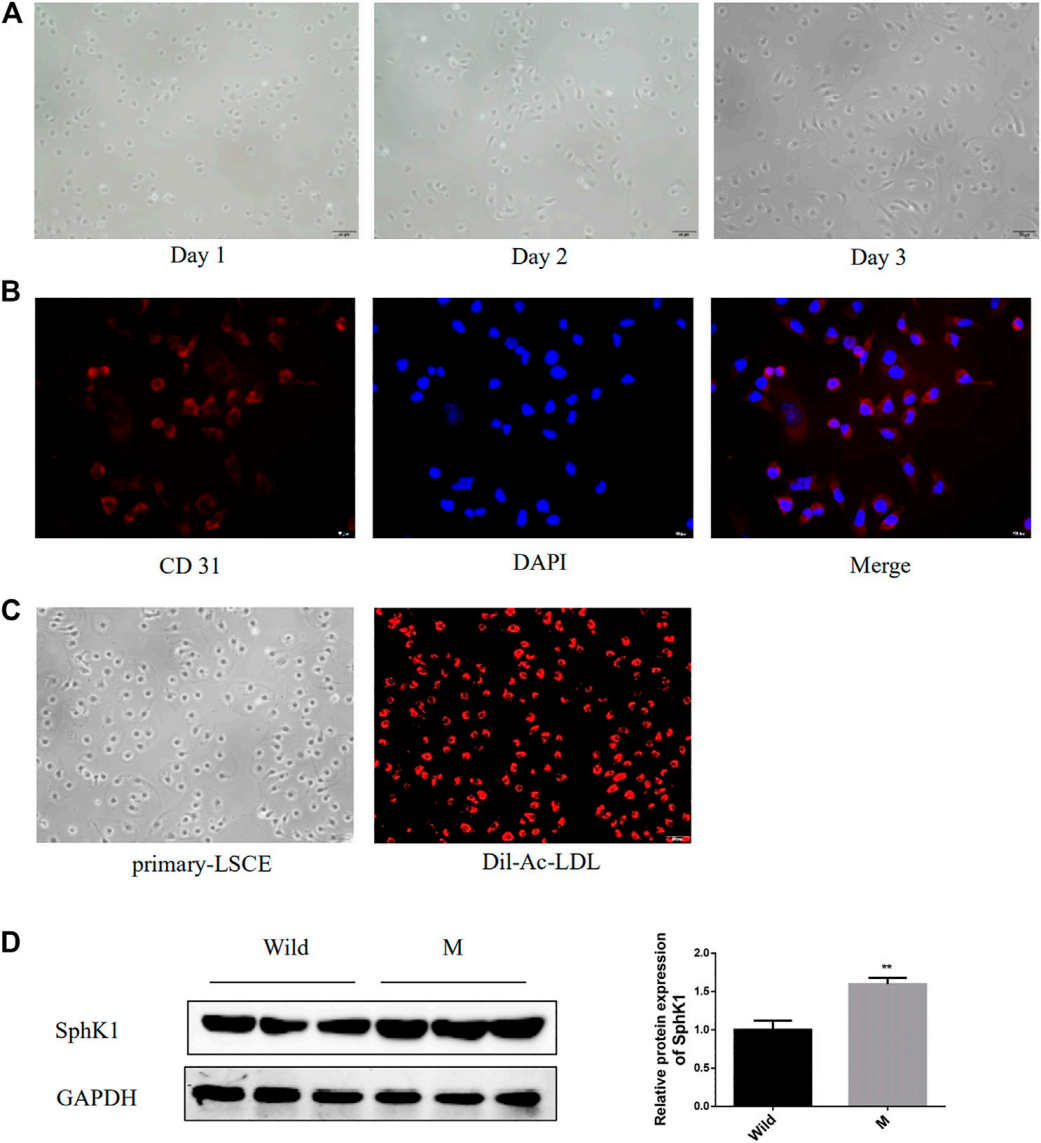
Expression of SphK1 in LSECs isolated from mice with liver fibrosis induced by CCl_4_. **(A)**: Morphology of isolated mouse primary LSECs. **(B)**: Isolated mouse primary LSEC could phagocytosis Dil-Ac-LDL. **(C)**: Isolated mouse primary LSECs expressed CD31. **(D)**: LSEC of mice with liver fibrosis upregulated the expression of SphK1 (*n* = 5, **p < 0.01, vs. Wild.)

Serum-derived exosomes contain too many contents, not limited to a certain organ or even a certain type of cell. Therefore, it is very difficult to trace whether the components in serum exosomes originate from a certain cell. Considering the special anatomical position of LSEC and HSC and the essential role of the interaction between LSECs and HSC in the process of liver fibrosis (Deleve, 2015), we cultured the primary LSEC and detected its expression of SphK1, trying to prove the source of SphK1. The freshly isolated LSECs were small and spherical. During the *in vitro* culture, the LSECs were observed to gradually stretch out and adopt a long spindle shape (Figure 5A). Figures 5B,C present isolated LSECs phagocytosed Dil-Ac-LDL and expressed CD31, both of which are features of LSECs. Primary LSECs were also isolated from liver fibrosis model mice prompted by CCl_4,_ which expressed considerably higher SphK1 than that in the Wild mice (*p* < 0.01) (Figure 5D).

### Sal Alleviated Exosomal SphK1-Induced LX-2 Migration and Activation by Inhibiting AKT Activation

**FIGURE 6:**
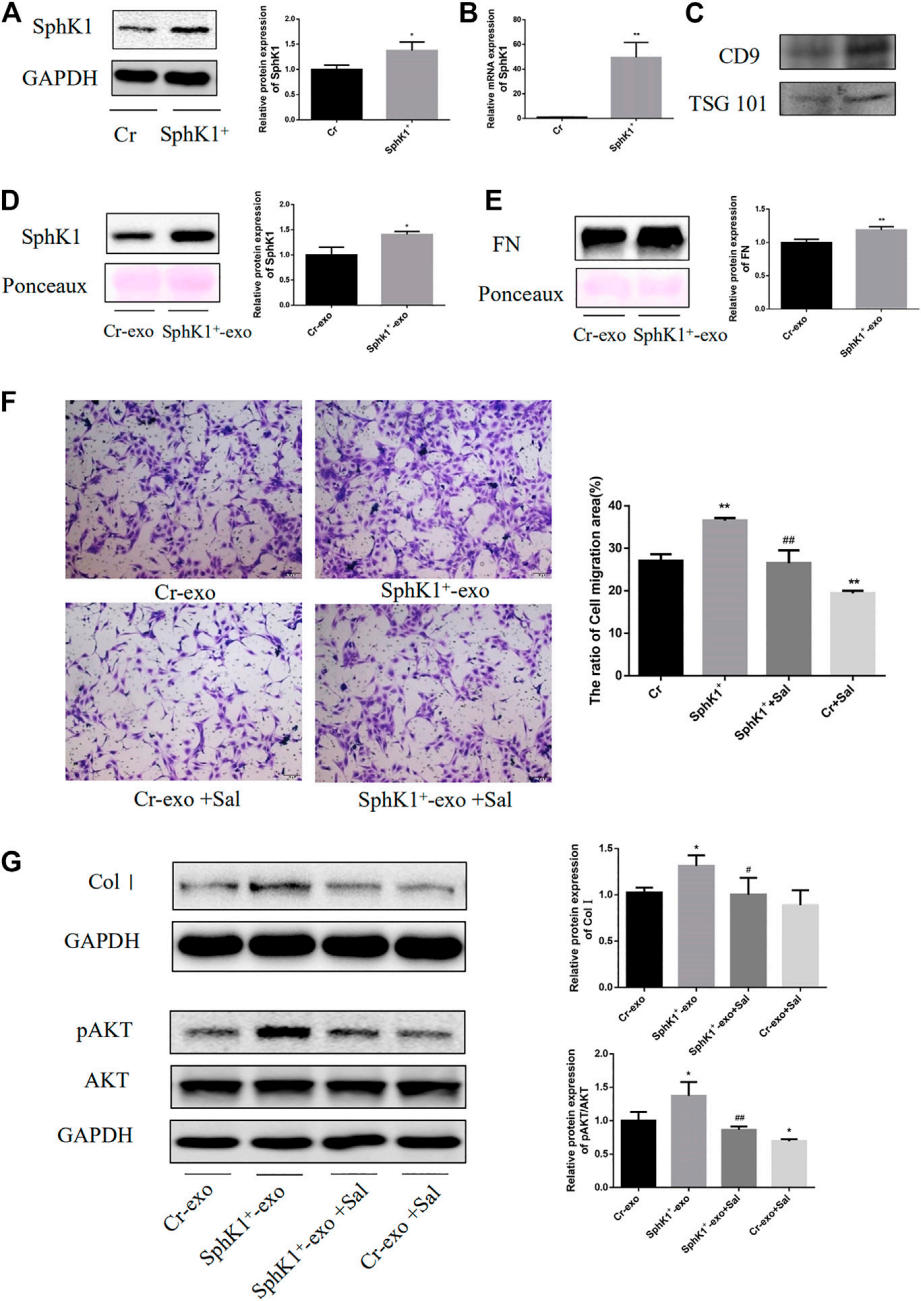
Effects of Sal on exosomal SphK1-induced LX-2 migration and activation. **(A)**: Expression of SphK1 in transfected cells was detected by WB. **(B)**: Expression of SphK1 in transfected cells was detected by PCR. **(C)**: Expression of TSG101 and CD9 in exosomes from culture mediums. **(D,E)**: Expression of SphK1 and FN in exosomes from culture mediums of transfected cells. (*n* = 3, **p < 0.01, vs Cr-exo.) **(F)**: Sal inhibited exosomal SphK1-induced LX-2 migration and activation. **(G)**: Sal inhibited exosomal SphK1-induced AKT phosphorylation in LX-2. (*n* = 3, *p < 0.05, **p < 0.01, vs. Cr-exo; ^#^ *p* < 0.05, ^##^ *p* < 0.01, vs. SphK1^+^-exo.).

High levels of SphK1 were detected in primary mouse LSECs of liver fibrosis mice. Although the effect of exosomal SphK1 was tried to be studied on LSECs and HSC, the exosomes could not be derived owing to the inadequate number of primary LSECs. Thus, SK-HEP-1 cell lines that function similarly to LSECs were used (Cogger et al., 2008). Overexpression SphK1, SK-HEP-1, was established, and exosomes were isolated from the culture medium to study how exosomal SphK1 influenced the function of LX-2. As displayed in Figures 6A,B, substantially protein and mRNA expression of SphK1 was detected in SK-HEP-1 cells transfected with the lentivirus carrying SphK1 as compared with empty vector-transfected SK-HEP-1 cells (*p* < 0.05 or *p* < 0.01) (Figures 6A,B).

As predicted, the exosomes from the culture medium of SphK^+^-SK-HEP-1 cells released significant quantities of SphK1. Moreover, upregulation of FN was recorded in the exosomes of SphK^+^-SK-HEP-1 cells (*p* < 0.05 or *p* < 0.01) (Figures 6D,E). LX-2 cells were incubated with exosomes to explore the influence of exosomal SphK1 on the function of LX-2. As displayed in Figure 8F and G, the exosomal SphK1 caused the migration of LX-2 and the Col I level in LX-2, which could be reversed by Sal (*p* < 0.05 or *p* < 0.01) (Figures 6F,G). Earlier studies have evidenced AKT phosphorylation to support HSC activation, while exosomal SphK1 was found to encourage AKT activation, and Sal reduces this effect in this research (*p* < 0.05 or *p* < 0.01) (Figure 6G). Thus, Sal was theorized to lessen LX-2 migration and activation induced by exosomal SphK1 by hindering AKT activation.

## Discussion

According to the complex pathological mechanism of fibrosis, multiple targets and pathways are the traits of traditional Chinese medicine. Traditional Chinese herbal medicine has been increasingly substantiated to treat liver fibrosis (Li, 2020), as it has effectively established the protective influence of Sal on liver fibrosis (Wu et al., 2003; Feng et al., 2018). Sal has already been proved to inhibit the migration of HSC in one of our earlier researches (Zhang et al., 2012). Exosomes have been explored extensively in recent years for their vital function as carriers for drug delivery. Earlier studies have revealed curcumin-encapsulated exosomes to possess anti-inflammatory, antioxidant, and anticancerous properties (Oskouie et al., 2018). In this study, a mouse model of liver fibrosis triggered by CCl_4_ was replicated further to investigate Sal's potential influence on liver fibrosis. Exosomes carrying SphK1 secreted by SK-HEP-1, a cell line with endothelial cell function, were also isolated, and their effect on HSC function and the effect of Sal treatment *in vitro* was analyzed.

Inflammation is a significant feature of fibrosis; constraining hepatic inflammation is essential in treating liver fibrosis. Previous studies have reported that Sal reduces liver fibrosis through anti-inflammatory (Feng et al., 2018). In this study, Sal was not just found to efficiently reduce the serum concentrations of ALT and AST in CCl_4_-induced liver fibrosis mice but also to hinder the infiltration of inflammatory cells into the liver tissue, evidencing that Sal could ease the damage caused by inflammation in the process of liver fibrosis. Unfortunately, in order to reserve as much serum as possible for the extraction of exosomes, this study did not detect IL1, IL6, TNF-α and other inflammatory factors in mouse serum, and failed to explain the anti-inflammatory effect of Sal more intuitively.

Cell apoptosis is known to have an essential role in embryonic development and tissue homeostasis (Tuzlak et al., 2016), but apoptotic cells can activate a series of pathological events under certain circumstances (Schwabe and Luedde, 2018). Apoptosis of damaged hepatocytes amplified inflammation, HSC activation, and liver fibrosis (Kisseleva and Brenner, 2020). Cell apoptosis primarily includes extrinsic and intrinsic pathways. The intrinsic or the mitochondrial-mediated apoptosis pathway is essentially regulated by the Bcl-2 family (Bock and Tait, 2020). Bcl-2 family is divided into 3 categories on the basis of function and structure: BH3, the apoptosis initiator; the antiapoptotic guardians, such as Bcl-2; and the proapoptotic proteins, such as Bax and Bak (Czabotar et al., 2014). Various cytotoxic stimuli activate BH3 pathologically, subduing the expression of antiapoptotic proteins and activating proapoptotic proteins, thus destroying the permeability of the mitochondrial inner membrane. Mitochondrial apoptosis is known to be co-regulated by the Bcl-2 family; the ratio of Bcl-2 to Bax is the key determining factor of apoptosis (Yu et al., 2021). Damaged mitochondria secrete cytochrome C that in turn is known to activate the caspase family, which is a group of cysteine protease that participates in cell apoptosis (Van Opdenbosch and Lamkanfi, 2019). Of these, caspase-3 is the central link and an essential initiator of the apoptosis process (Galluzzi et al., 2016). Caspase-3 physiologically exists as an inactive zymogen. Caspase-3 is rapidly cleaved and activated to trigger cell death signals in case of various injuries (Lim et al., 2021). In the present study, the hepatocyte apoptosis was observed in the Model group, the expression of cleaved caspase 3, cleaved PARP and Bax were upregulated, while Bcl-2 was downregulated. Sal was observed to reverse the high expression of Bax and low expression of Bcl-2 induced by CCl_4_ and upregulate the ratio of Bcl-2 to Bax. Sal was also observed to inhibit the activation of caspase-3 and PRAP, the substrate of caspase-3, thereby hindering cell apoptosis.

SphK1 has been shown to be involved in the regulation of mitochondrial permeability transition by several studies. Besides, SphK1 deficiency hinders JNK activation and defends acetaminophen-induced hepatocyte death (Li et al., 2019), whereas SphK1 overexpression was found to aggravate cell apoptosis (Lu et al., 2018). In this study, Sal was detected to alleviate hepatocytes apoptosis as well as JNK activation. The effect of Sal on the SphK/S1P/S1PRs signaling pathway in CCl_4_-induced hepatic fibrosis mice was also explored. S1P, bioactive sphingomyelin, is released by Sphingosine kinases (SphKs) catalyzing Sphingosine, which participates in regulating various biological activities such as cell proliferation and migration. Two subtypes of SphK are SphK1 and SphK2 (Ogretmen, 2018). SphK1 can migrate from the cytoplasm to the plasma membrane and promote S1P formation on the cell membrane under the influence of growth factors, hormones, or cytokines. Furthermore, SphK1 occurs chiefly in the cytoplasm, whereas SphK2 in the nucleus and mitochondrial inner membrane, which can likely upsurge the S1P concentration in the nucleus (Spiegel and Milstien, 2011). High quantities of SphK1 expression have been shown recently to aid in the progression of liver fibrosis (Sato et al., 2016), whereas SphK1 inhibition substantially reduces the degree of liver fibrosis (Yang et al., 2013). Another study found compared with that in healthy volunteers, the level of SphK2 was reduced in the liver of patients with alcoholic cirrhosis and hepatocellular carcinoma, and SphK2 deficiency could aggravate liver injury induced by alcohol (Kwong et al., 2019). Our date suggested that the expression of SphK1 in the fibrotic liver was remarkably upregulated, and the SphK2 level was obviously reduced, in agreement with the findings of earlier researches. While Sal could reverse these changes, whether these changes were stimulating or hindering. Moreover, there are growing researches regarding of the expression level of S1P and its ligands and their roles in liver fibrosis. For instance, Yang et al. (Yang et al., 2013) reported the expression of S1P increased progressively with the aggravation of liver fibrosis in mice induced by CCl_4_ or BDL. Besides, their study displayed these changes of S1P are not only manifested in fibrotic animals but also upregulated in liver tissues from hepatitis B, C, and alcoholic cirrhosis patients (Yang et al., 2013). In this study, high concentrations of S1P were also found in the mice serum with liver fibrosis and Sal reversed the change of S1P. Earlier studies have established that S1PR1, S1PR2, and S1PR3 can be expressed in human liver fibroblasts (hepatic myofibroblast, hMFs). Remarkably, S1PR1 and S1PR3 occurred in high concentrations in fibrotic liver tissues, whereas S1PR2 expression was downregulated (Li et al., 2011), the findings being in agreement with those of our experiments. Essentially, Sal reversed the increase in S1PR1 and S1PR3 and the decrease in S1PR2 induced by CCl_4_. Based on published research, the differences in the SphK/S1P/S1PRs signaling pathway among humans, rats, and mice samples may be owing to the species differences and diverse fibrogenesis mechanisms. Additional studies are indispensable to investigate the role of the SphK/S1P/S1PRs signaling pathway in regulating liver fibrosis and its therapeutic significance.

After observing the effects of Sal on SphK1 and S1PRs *in vivo*, we tried to clarify whether the influence of Sal on SphK1 and S1PRs in liver tissue could be localized on certain cells. Considering the key role of HSC in liver fibrosis, LX-2 was treated with PMA (an agonist of SphK1) and/or Sal. The results showed that Sal could inhibit HSC migration induced by PMA, but did not affect the expressions of SphK1 and S1PRs in LX-2 (Data not shown). Combined with previous studies reported that exosomal SphK1 derived by LSEC could induce HSC migration (Wang et al., 2015). Therefore, we tried to illustrate effect of Sal on HSC based on regulation of LSEC exosomes on HSC.

Exosomes are extracellular vesicles of diameters ranging from 50 to 150 nm (van Niel et al., 2018). They possess the same characteristics containing “exosome marker” proteins related to exosome biogenesis and secretion, such as the Rab family, GTPase, ALG-2-interacting protein X (ALIX), and TSG101. Exosomes are also rich in heat shock proteins, such as HSP60, HSP70, and HSP90; integrins; and tetraspanins, viz, CD9, CD63, CD81, and CD82. However, the inclusions of exosomes are highly heterogeneous based on the state and type of cells (Sung et al., 2018). For instance, the expression of CD81 in serum exosomes of patients with chronic hepatitis C was significantly higher than that in the healthy volunteers and patients in the virus response phase (Martin-Walter et al., 2012). The exosomes of lipid-induced hepatocyte secreted high amounts of miR-128-3p, which stimulated HSC migration after being taken up by HSC(Povero et al., 2015). In this study, serum exosomes derived from liver cirrhosis patients or mice with liver fibrosis released high concentrations of SphK1 and promoted HSC migration. Simultaneously, compared with serum-derived exosomes from Model group mice, serum-derived exosomes from Sal group mice expressed lower SphK1 and reduced JS 1 migration. In addition, exosome-mediated intercellular communication has been shown to require docking on the plasma membrane in an earlier study (Teng and Fussenegger, 2021). Also, integrins, lipids, heparan sulfate proteoglycans, and ECM components (especially FN and laminin) can function as mediators in the intercellular interaction (van Niel et al., 2018). In this study, the expression of FN was upregulated in exosomes of SphK^+^-SK-HEP-1 cells, signifying that the FN on the surface of exosomes mediated interactions between exosomes and cells thus influencing the function of the recipient cells. The concentration of SphK1 was amplified in primary LSECs of liver fibrosis mice. Also, exosomal SphK1 derived from SK-HEP-1, a cell line with a similar function to that of LSECs, promoted the migration, activation, and AKT phosphorylation of LX-2. Sal relieved these effects. These outcomes indicated that Sal likely alleviated the migration and activation of LX-2 induced by exosomal SphK1 by inhibiting the activation of AKT.

To conclude, our study evidenced that Sal could effectively reduce liver injury, hepatocyte apoptosis, and liver fibrosis *in vivo*. In addition, the protective efficacy of Sal might be comprehended by regulating JNK activation and controlling the SphK/S1P/S1PRs axis. Moreover, Sal was observed to reduce the migration and activation of LX-2 induced by exosomal SphK1 by subduing the activation of AKT *in vitro*. In summary, the present study explained the anti-fibrosis mechanism of Sal based on the perspective of regulating the biological activity of HSC by LSEC-derived exosomes. More experiments are needed to further explain the role of Sal on liver fibrosis.

## Data Availability

The original contributions presented in the study are included in the article/Supplementary Material, further inquiries can be directed to the corresponding authors.
